# Bullying victimization CBT: a proposed psychological intervention for adolescent bullying victims

**DOI:** 10.3389/fpsyg.2023.1122843

**Published:** 2023-08-22

**Authors:** Louise Ferraz De Camargo, Kylie Rice, Einar Baldvin Thorsteinsson

**Affiliations:** School of Psychology, University of New England, Armidale, NSW, Australia

**Keywords:** adolescents, bullying, CBT, mental health, psychological, treatment, victimization

## Introduction

Bullying is a prevalent societal concern with one in three children being victims of bullying globally (UNESCO, 2019). Bullying during adolescence is associated with a comprehensive cluster of symptoms including loneliness, suicide ideation and intent (Moore et al., 2017), depressive symptomology (Ferraz de Camargo and Rice, 2020), generalized anxiety, social anxiety, and more recently separation anxiety, panic disorder, and obsessive-compulsive disorder symptomology (Ferraz de Camargo et al., 2022). Bullying victimization is associated with reduced cognitive flexibility and emotional regulation capacity (Palamarchuk and Vaillancourt, 2022), and behavioral issues (Idsoe et al., 2021). Many of these outcomes may persist into adulthood (Moore et al., 2013, 2017).

Investigation of the literature to date suggests that efforts to reduce the negative effects of bullying on adolescent mental health have focused on reducing bullying behavior, and that this approach has had limited success (Gokkaya, 2017; Menesini and Salmivalli, 2017; Jadambaa et al., 2020). Further, treatment for the effects of bullying typically often occurs in school group settings (Gokkaya, 2017). By comparison, research investigating specific psychological treatment that directly supports the individual victim has been neglected. The present paper aims to address this by investigating existing evidence that supports the adoption of the cognitive behavioral therapy (CBT) model (Beck, 1976) as a potential framework for psychological treatment targeted at the sequelae of psychological issues associated with bullying victimization. BV-CBT takes a developmental perspective and considers the impact of victimization during the developmental stage of childhood and adolescence. It is hoped that future research will build on this proposal and test this model through appropriately designed studies.

### Bullying defined

Bullying comprises of three key aspects: (1) intent to harm; (2) repeated over time; and (3) an imbalance of power between the perpetrator and the victim. Thus, bullying refers to ongoing, aggressive, unwanted and unjustified behavior, that the victim feels powerless to combat (Olweus, 1978). This definition has continued to be adopted by researchers internationally (UNESCO, 2019). Types of bullying include verbal, physical, and social and can occur in different settings. Verbal, physical, and social bullying can occur in person. Verbal and social bullying can occur online which is often referred to as cyber bullying (Cross et al., 2009). Traditional bullying refers to more easily observed *overt* behavior including being hit, kicked, or having personal belongings stolen or destroyed while *covert* bullying refers to subtle yet aggressive, disguised, non-physical behavior that is hidden from teachers, parents, and other adults (UNESCO, 2019). For example, peer relationships can be used to inflict harm through social exclusion, ignoring the victim, or the spreading of malicious rumors, with the aim to destroy self-esteem and sense of belonging and acceptance (Cross et al., 2009). Covert types have been found to be equally or more psychologically damaging compared to overt bullying (Baldry, 2004; Ferraz de Camargo and Rice, 2020), at times resulting in social and psychological scars that may continue into adulthood (Crick and Bigbee, 1998; Prinstein et al., 2001; Archer and Coyne, 2005).

### The whole-school approach: limitations

Currently, efforts to curb the mental health outcomes associated with bullying victimization utilize the whole-school approach. This approach takes a socio-ecological perspective that involves governments, school communities, and families working together to implement educational and anti-bullying programs (Cross et al., 2011; Evans et al., 2014). However, while reducing bullying behavior is desirable, this may fall short of supporting individual victims.

For example, a systematic and meta-analytic review investigating effectiveness of programs focused on 12 countries across three regions (i.e., North America, Europe, and Scandinavia) and identified sixty-five different school-based bullying intervention and prevention programs aimed at reducing bullying behavior and victimization (Gaffney et al., 2019). Crucially, the majority of these programs had not undergone repeated evaluation and implementation more than once using independent samples. Indeed, only four of the sixty-five programs had been evaluated more than twice across different locations and with different evaluators; KiVa, NoTrap!, OBPP, and ViSC (Gaffney et al., 2019).

Considering the four programs combined, the whole-school approach was somewhat effective with perpetration and victimization reduced by ~19–20% and 15–16% respectively, thus suggesting both perpetration and victimization remain prevalent. However, results suggest that the whole-school approach is not always the most effective, and might not be effective for every individual student. For example, the KiVa program was “marginally effective” at ~11% decrease in victimization, and although the effect sizes of the ViSC program were not statistically significant, the odds ratios correspond to an increase in victimization by ~4%. Determining these variations in effectiveness is complicated with influencing factors including the country, region, or cultural setting, and the location and population for which the program was initially developed (Gaffney et al., 2019). Additional factors to consider are the intensity and duration of the program, teacher training, students' age, cultural background, and parental involvement. Moreover, the specific components that contribute, or not, to desired outcomes remain unclear (Menesini and Salmivalli, 2017). Indeed, the effectiveness of the whole-school approach is often questioned with researchers concluding that programs vary from somewhat effective to not at all, and in some circumstances to increased victimization and exclusion (Vreeman and Carroll, 2007). In conclusion, Gaffney et al. (2019) suggest that the whole-school approach may not be the best strategy to combat bullying perpetration and victimization and that targeted interventions to help individual children are needed.

Regarding cost-effectiveness, calls have been made for high-quality evaluations to provide evidence to determine the components of the whole-school anti-bullying approach that reduce perpetration and victimization (Vreeman and Carroll, 2007; Menesini and Salmivalli, 2017). Quite surprisingly, the lack of evidence underpinning anti-bullying programs and the whole school approach is reflected in the implementation of the Australian National Safe Schools Framework as a national initiative without any longitudinal empirical evaluation of the program's effectiveness (Cross et al., 2011). Notably, the enthusiastic adoption of the whole-school approach and its inclusion into national law in some countries has been suggested to be based on the desperate need to take action rather than on clear evidence of effectiveness (Smith et al., 2004).

### Bullying victimization: a chronic, developmental trauma

Shifting the conceptualization of bullying victimization from a social phenomenon to a chronic, developmental trauma emphasizes the need for the development of psychological treatment for victims. The links between bullying and post-traumatic stress disorder (PTSD) have been investigated in a literature review and meta-analysis of 29 cross-sectional studies on the relationship between workplace and school bullying with PTSD symptomology (Nielsen et al., 2015). Results demonstrated a significant association between bullying and an overall symptom-score of PTSD, as well as significant correlations between bullying and specific PTSD symptoms, for both adults and children. Additionally, results indicated that 57% of victims reported symptoms of PTSD above clinical levels (Nielsen et al., 2015).

Indeed, as shown in Table 1, according to the Diagnostic and Statistical Manual of Mental Disorders Fifth Edition (DSM-5, 2013), post-bullying mental health outcomes (see Moore et al., 2017 for an overview) typically meet the diagnostic criteria for PTSD (American Psychiatric Association, 2013). However, it should be noted that bullying takes on many forms and is not limited to threats of death or injury, as such, Criterion A may not always be met. This presents a barrier to bullying victims receiving a diagnosis of PTSD, thus inhibiting diagnosis and appropriate psychological treatment.

**Table 1:** Comparison of DSM-5 PTSD criteria and bullying victimization outcomes.

**DSM-5 PTSD criteria**	**DSM-5 criterion summary**	**Bullying victimization outcomes**
Criterion A: stressor	Exposure to: death, threatened death, actual or threatened serious injury, or actual or threatened sexual violence	Victims typically feel threatened, although not all bullying will meet the full criterion A.
Criterion B: intrusion symptoms	The traumatic event is persistently re-experienced	Bullying victims experience intrusive, distressing memories of the event/s
Criterion C: avoidance	Avoidance of trauma-related stimuli after the trauma	Avoidance of the school environment and friends as seen in school absenteeism is common in victims
Criterion D: negative alterations in cognitions and mood	Negative thoughts or feelings that began or worsened after the trauma	Anxiety, depression, shame, self-blame, feeling emotionally numb, loss of interest in usual activities, suicide ideation and intent
Criterion E: alterations in arousal and reactivity	Trauma-related arousal and reactivity that began or worsened after the trauma	Victims may experience irritable behavior, self-destructive behavior such as self-harm, difficulty concentrating, difficulty with self-regulation, sleep issues, anxiety, depression
Criterion F: duration	Symptoms last for more than 1 month	Symptoms can persist into adulthood
Criterion H: exclusion	Symptoms are not due to medication, substance use, or other illness	Detrimental mental health outcomes have been shown to be uniquely related to bullying victimization

Building on this, a recent review of the literature suggests that the outcomes of bullying are more complex than that of PTSD symptomology. Indeed, the review demonstrated that a developmental perspective should be adopted in the conceptual understanding of the negative outcomes associated with being bullied. The authors conclude that reactions to being bullied are better understood as a combined framework of a developmental trauma disorder and a complex post-traumatic disorder as bullying is experienced over time, in some cases for years, at a time when cognitive capacity continues to develop (Idsoe et al., 2021). Indeed, the developmental aspect of bullying is a key differentiating factor from trauma related to PTSD which, according to the DSM-5, is typically related to single event (American Psychiatric Association, 2013). Supporting this are recent findings that bullying during childhood results in neuroendocrine reactivity that negatively impacts emotion processing and executive functioning such as semantic cognition, cognitive flexibility, and learning. This influences sensitivity to facial expressions, poor cognitive reasoning, and distress which then impacts behavioral modulation and emotion regulation (Palamarchuk and Vaillancourt, 2022). These findings underpin the importance of psychological intervention that supports victims to increase their cognitive flexibility and their ability to regulate their emotional and behavioral responses.

The need to understand childhood trauma within a developmental psychopathological perspective was stressed by van der Kolk et al. (2009) proposal for the DSM-5 (American Psychiatric Association, 2013) of a new diagnosis coined “Developmental Trauma Disorder (DTD)”. Although the proposal was not accepted by the DSM committee, a similar diagnosis “complex post-traumatic disorder” was introduced into the International Classification of Diseases-11 (World Health Organisatio, 2019). van der Kolk et al. (2009) argued that it is clear that a diagnosis would clarify the presentation of children and adolescents exposed to the chronic trauma of bullying victimization during this developmental stage and offer guidance to treating clinicians and support the development of effective interventions. It would also help combat victims receiving multiple, unrelated diagnoses (van der Kolk et al., 2009). Indeed, calls have also been made for the development of diagnostic criteria for “post-bullying” disorder that encapsulates the complex cluster of symptoms associated with bullying victimization in the hope of increasing screening, diagnosis, and treatment by psychologists and other mental health professionals (Arnout et al., 2019).

In sum, while a specific diagnosis and diagnostic criteria is yet to be established for bullying victimization, appropriate treatment is needed to address the well-evidenced associated mental health issues. Psychological intervention for bullying victims needs to take a holistic, developmental approach and address the cognitive, behavioral, and emotional components in order to support victims to manage bullying in a manner that is more protective to mental health. This is in contrast to treating bullying victims' experience of a particular mental health concern, such as anxiety, in isolation.

### BV-CBT: a proposed model

A systematic review of the literature published from 2012 to 2022 was conducted to ascertain the existence of established psychological evidence-based, individualized treatment for cognitive, behavioral, and emotional challenges experienced by bullying victims. The Preferred Reporting Items for Systematic Reviews and Meta-Analysis (PRISMA) guidelines were followed. Multiple electronic databases were systematically searched including EBSCOHost, ERIC, Informit, ProQuest Psych, PsychArticles, PsychInfo, and Scopus. Additionally, a manual search of reference lists from relevant reviews was done. The following search terms were used in varying combinations to perform an ABTI search: psychotherapy, therapy, intervention, bullying victim, adolescents, teens, youth, peer. Eligible articles included English-language papers published in peer-reviewed journals. Inclusion criteria were: adolescents aged 12–18 years who were victims of bullying, individualized, evidence-based psychological treatment for victims of bullying. Studies published in a language other than English were excluded.

Based on the results of this systematic search, no individualized, evidence-based psychological interventions specifically for mental health issues associated with bullying victimization were identified.

Considerations for determining a bullying victimization intervention model are that the intervention must have proven effective for the cluster of symptoms commonly experienced by bullying victims previously outlined (e.g., cognitive flexibility, emotional regulation, behavioral issues, and fear responses). Existing research suggests that the CBT model (Beck, 1976) offers a well-established, theoretical, evidence-based framework in which these issues can be understood and treated within a developmental perspective (Ferraz de Camargo and Rice, 2020; Ferraz de Camargo et al., 2022).

According to guidelines developed by the National Institute for Health and Care Excellence (NICE, 2020) and by the Royal Australian and New Zealand College of Psychiatrists (RANZCP; Andrews et al., 2018), CBT is considered first-line treatment for a vast array of psychological disorders, including suicide ideation and intent (Hua et al., 2022), all types of anxiety, depressive presentations, emotional regulation, cognitive flexibility, and behavioral issues, all of which are common outcomes of bullying victimization (Moore et al., 2017; Idsoe et al., 2021; Ferraz de Camargo et al., 2022). CBT has long been regarded as an effective therapy for managing the influence of unhelpful thoughts on emotional distress and physiological responses in relation to a given situation through increasing psychological flexibility and combating avoidance behavior (Beck, 1976).

In the case of bullying, the experience of the negative life event alone does not explain negative psychological outcomes (Moore et al., 2017). Rather, it is the cognitive interpretation of the situation that influences internalizing and externalizing problems (Moore et al., 2017). Considering specific bullying tactics such as physical aggression, social exclusion by close friends, or having one's reputation ruined, it is not surprising that victims experience distorted cognitions that increase risk for internalizing problems including anxiety, depression, and suicide ideation or intent (Kearney, 2001).

CBT improves mental health outcomes by supporting individuals to identify, evaluate, and reframe their distorted cognitions, with techniques such as evidence-based thinking and introducing helpful cognitive coping strategies (Courtney et al., 2011). CBT also aims to increase helpful behavior through behavioral activation techniques such as activity scheduling and exposure therapy. These strategies produce more helpful behavior and emotions which reinforces new, more positive cognitions, thus reversing the cycle (Beck, 1976; Courtney et al., 2011). For example, adopting more helpful ways of viewing the bullying situation may encourage behavior modification such as increasing engagement with friends and attending school. This would then provide evidence to support new, helpful cognitions such as “I belong”, “I have friends I can count on”, and “I can manage this situation”. Indeed, CBT is well-recognized for increasing cognitive flexibility, encouraging increased interpersonal interaction and reducing avoidance behavior, all of which are key to supporting bullying victims.

Considering the developmental perspective, Palamarchuk and Vaillancourt (2022) explain that children and adolescents are more vulnerable to the chronic stress of bullying victimization in comparison to adults due to neurobiological responses that could result in psychopathology. This is based on the stressor occurring at a time when neuronal development is occurring. Further, the authors reinforce Moore et al. (2017) view that it is the cognitive appraisal, or the interpretation of the stressor rather than the nature of the stressor itself, that determines the perceived severity and controllability of the threat of the bullying event. Thus, reducing the perceived threat and increasing perceived controllability is fundamental.

Although the skill of using cognitive coping strategies, which is fundamental to psychological flexibility, is still being mastered during the developmental stage of adolescence with full acquisition being achieved in adulthood (Garnefski and Kraaij, 2006; Zimmer-Gembeck and Skinner, 2020), teaching these skills can be achieved through BV-CBT. Indeed, research has demonstrated that higher use of helpful cognitive coping strategies among adolescent bullying victims is associated with improved mental health outcomes. For example, Ferraz de Camargo and Rice (2020) found that the helpful cognitive coping strategy ‘positive reappraisal' moderated the relationship between bullying victimization and depressive symptomology. Further, Garnefski and Kraaij (2014) demonstrated that ‘rumination' strengthened and ‘positive refocusing' reduced victims' experience of depression, while ‘rumination' and ‘catastrophising' strengthened and ‘positive reappraisal' reduced victims experience of anxiety. Further, a retrospective study found that group-based CBT reduced self-reported anxiety symptomology and reports of perceived victimization among 6–17-year-olds (Hunt et al., 2022). These findings offer support for the theory underlying the BV-CBT model and the proposal that through CBT, symptoms of bullying victimization can be combated.

**Figure 1:**
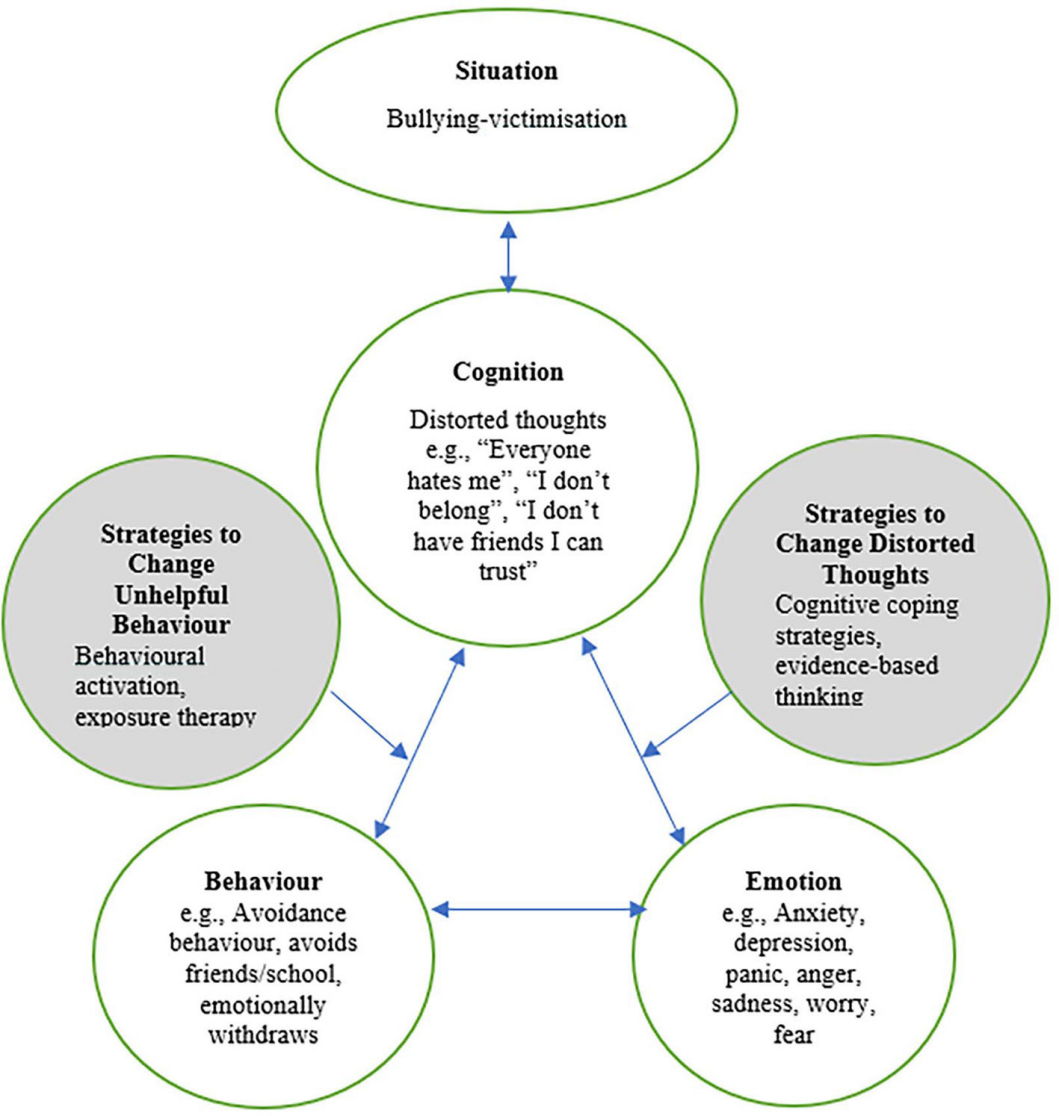
Proposed “cognitive-behavioral model of bullying-victimization” and hypothesized CBT-based strategies to combat unhelpful cognitions and behavior in relation to bullying victimization.

Bullying victimization has long been associated with generalized anxiety disorder and social anxiety disorder (Hawker and Boulton, 2000). However, recently the common component of fear was explored revealing that victimization is also associated with other anxiety subtypes including panic disorder, separation anxiety, and obsessive-compulsive disorder symptomology (Ferraz de Camargo et al., 2022). Considering that bullying victimization is now recognized as underlying many anxiety presentations during adolescence (Hawker and Boulton, 2000; Moore et al., 2017; Stanaway et al., 2018), modifying unhelpful thoughts and behavior in relation to bullying victimization through BV-CBT at the developmental stages of childhood and adolescence may result in improvements in a range of mental health outcomes. In conclusion, this paper proposes that the well-established effectiveness of CBT could be applied to improve mental health outcomes for victims of bullying through a new BV-CBT model that adopts a holistic, developmental approach targeted at individualized treatment. The proposed model is presented in Figure 1.

## Discussion

The results of the systematic review demonstrate that a framework for evidence-based psychological intervention that supports individualized treatment for victims of bullying is lacking. The concerns expressed by van der Kolk et al. (2009) that victims are treated for multiple, unrelated mental health issues is warranted. Without a bullying victimization model, anxiety, depression and other mental health issues experienced by bullying victims will continue to be diagnosed and treated in an isolated manner without considering or treating the constellation of psychosomatic and developmental issues commonly experienced by victims.

Decades have been spent focusing on reducing bullying behavior as a means to reducing the negative consequences experienced by victims. While some reduction in bullying behavior may have been achieved, bullying behavior is still prevalent and many victims continue to suffer. Governments, teachers, parents, students, and school communities are working together across the world to implement whole-school anti-bullying approaches. However, effectiveness is impacted by the complexity and number of components that are required to be implemented systematically and consistently by several parties. Certainly, there is a lack of high quality studies to determine the effectiveness of these programs and calls have been made for this to be rectified (Gaffney et al., 2019).

### Future direction

Future research is needed to identify the unique, complex characteristics, unique to bullying victimization. These investigations can inform the conversation around the diagnostic criteria for bullying victimization and will help combat victims meeting criteria for multiple diagnosis which is a barrier to holistic treatment (van der Kolk et al., 2009; Idsoe et al., 2021). Additionally, testing of the model is needed to evaluate the effects of BV-CBT on the cognitive, behavioral, social, and developmental outcomes for bullying victims. Supporting this future research is the proposed BV-CBT model which offers a framework for individualized, evidence-based, treatment for victims of bullying that adopts a developmental perspective.

Shifting the conceptualization of bullying victimization from a social phenomenon to chronic, developmental trauma experienced at the individual level leads to psychological intervention as being key to supporting victims. We can no longer only apply the socio-ecological perspective and wait for values and norms within schools and the wider culture to change in order for solutions to be achieved. It is time to also consider the individual, developmental perspective, and support victims with their bullying experiences in a way that protects mental health. Despite the potential for psychological intervention to improve mental health outcomes for bullying victims, a specific framework and psychological intervention for bullying victimization is lacking. In response, the present paper proposes the BV-CBT model in the hope of encouraging much needed holistic, evidence-based psychological intervention for bullying victims that adopts a developmental perspective. It is also hoped that BV-CBT may be used not only in the aftermath of bullying, but as a preventative intervention that may reduce the widespread and traumatic consequences of this chronic mistreatment for vulnerable adolescents. Considering the on-going prevalence and the serious, multiple, and potentially long-term negative effects of bullying on the developmental and psychological health of victims globally, rectifying this is urgent.

## Author contributions

LF conceived of the presented idea and wrote the draft of this article. KR provided insightful comments that critically improved the manuscript quality. KR and ET contributed to editing the manuscript. All authors contributed to the article and approved the submitted version.
